# Quantitative microbial risk assessment of Greywater on-site reuse

**DOI:** 10.1016/j.scitotenv.2018.04.197

**Published:** 2018-09-01

**Authors:** Kuang-Wei Shi, Cheng-Wen Wang, Sunny C. Jiang

**Affiliations:** a School of Environment, Tsinghua University, Beijing, China; b Civil and Environmental Engineering, University of California, Irvine, USA

**Keywords:** BOD, biochemical oxygen demand, pppy, per-person-per-year, DALYs, disability-adjusted life years, gpcd, gallons per capita per day, QMRA, quantitative microbial risk assessment, EAEC, Enteroaggregative *E. coli*, QMRA, Monte Carlo simulation, Pathogenic *E. coli*, Annual infection risk, Disease burden

## Abstract

Recycle domestic greywater for on-site non-potable uses can lessen the demand on potable water and the burden on wastewater treatment plants. However, lack of studies to assess health risk associated with such practices has hindered their popularity. A Quantitative Microbial Risk Assessment was conducted to estimate the public health risks for two greywater reuse scenarios: toilet flushing and food-crop irrigation. Household greywater quality from three sources (bathroom, laundry and kitchen) was analyzed. Mathematical exposure rates of different scenarios were established based on human behavior using Monte-Carlo simulation. The results showed that, greywater from all three household sources could be safely used for toilet flushing after a simple treatment of microfiltration. The median range of annual infection risk was 8.8 × 10^−15^–8.3 × 10^−11^ per-person-per-year (pppy); and the median range of disease burden was 7.6 × 10^−19^–7.3 × 10^−15^ disability-adjusted life years (DALYs) pppy. In food-crop irrigation scenario, the annual infection risks and disease burdens of treated greywater from bathroom and laundry (2.8 × 10^−8^, 4.9 × 10^−8^ pppy; 2.3 × 10^−12^–4.2 × 10^−12^ DALYs pppy) were within the acceptable levels of U.S. EPA annual infection risk (≤10^−4^ pppy) and WHO disease burden (≤10^−6^ DALYs pppy) benchmarks, while kitchen greywater was not suitable for food-crop irrigation (4.9 × 10^−6^ pppy; 4.3 × 10^−10^ DALYs pppy) based on these benchmarks. The model uncertainties were discussed, which suggests that a more accurate risk estimation requires improvements on data collection and model refinement.

## Highlights

• Health risks for two on-site uses of treated greywater are modeled.
• Food-crop irrigation exhibits a greater health risk than toilet flushing.
• Use of treated greywater for toilet flushing is within the acceptable health risk.
• Kitchen greywater is not recommended for food-crop irrigation.

## Graphical abstract

**Figure:**
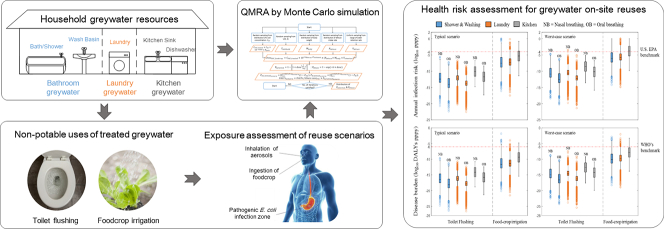

## 1 Introduction

In pace with the human population explosion, water shortage is regarded as one of the most concerned issues around the world. About 1 billion people worldwide now don't have access to safe drinking water (Connor, 2015). Development of new water resources is imperative. Instead of seawater desalination or drilling deeper into groundwater aquifers, collecting and recycling municipal wastewater for on-site non-potable uses can curtail demand of freshwater as well as lessen the burden of wastewater treatment plants. Such approach is likely to be a feasible and eco-friendly direction.

Municipal wastewater is generally divided into yellow water, brown water and greywater. Among them, yellow and brown water refer to urine and fecal sewage. Greywater, including streams from showers/baths, wash basins, laundry, kitchen sinks and dishwashers, is generally defined as urban wastewater without any pollution from toilets (De Gisi et al., 2016). Of these three, greywater is the most suitable for water reuse because of its large volume and low concentration of pollutants (DeOreo et al., 2016). The invention of source separation system makes it possible to reuse domestic greywater on-site as a new water supply (Larsen and Gujer, 1997). It segregates wastewater streams at their sources and treats them separately according to their qualities, so that water reuse can be more economical and less complicated.

Toilet flushing is one of the most frequently discussed application of greywater reuse, through which household indoor water demand can decrease by >20% (De Gisi et al., 2016; DeOreo et al., 2016; ENEA, 2002). Another common route of greywater reuse is outdoor irrigation (Lubbe et al., 2016; Pandey et al., 2014). Across many regions in the United States, the amount of outdoor water consumption accounts for >50% of total water use in a single household (DeOreo et al., 2016). Previous study indicated that greywater from bathroom and laundry not only can meet demands of toilet flushing entirely, but also can satisfy part of irrigation demand if necessary (National Academies of Sciences and Medicine, 2016).

Constituents in greywater are related to diverse factors, such as source of water supply, household activities and water-consuming installations (De Gisi et al., 2016). Generally, domestic greywater is generated from bathroom, laundry and kitchen; and their qualities can differ significantly (Li et al., 2009; Oron et al., 2014). Previous studies of greywater quality showed that bathroom greywater contains a low concentration of biochemical oxygen demand (BOD), nitrogen and phosphorous, and is regarded as the cleanest stream of greywater (Bodnar et al., 2014; Li et al., 2009; Oron et al., 2014). Due to the addition of laundry detergents, laundry greywater has an elevated alkalinity, pH and high loads of sodium, nitrogen, phosphorous and surfactants, but its level of BOD is relatively low. In contrast, kitchen greywater, which comprises oils, fats and food debris, shows high concentrations of BOD, nitrogen, phosphorous as well as turbidity, and is considered as the most polluted greywater. Despite the absence of urine or fecal contaminations, all streams of greywater, however, contain microbial contaminants. Even in bathroom greywater, the concentration of total coliforms can exceed 1 × 10^7^ CFU/100 ml (De Gisi et al., 2016), which poses a potential health threat during its practical reuse application.

Diverse reuse routes can result in different exposure scenarios of greywater to human bodies, which ultimately determine the magnitude of health risk. Toilet flushing and outdoor irrigation represent two distinct routes of greywater exposure: respiratory tract vs. digestive tract. For toilet flushing, greywater aerosols are produced after a single toilet flushing. The aerosols can be inhaled into human respiratory system, which brings in harmful constituents simultaneously (Lim et al., 2015). As for irrigation, it's been estimated that 31% of U.S. households participated in food-crop irrigation (Butterfield, 2009). The intake of raw home-grown produce provides a direct pathway for greywater retained on crops to enter human bodies (Lim and Jiang, 2013). Although exposure to hazards doesn't necessarily equal an unacceptable threat, such facts do indicate the existence of potential risks.

Previous studies claimed that untreated (or inadequately-treated) greywater is not recommended for either potable or non-potable uses (Etchepare and van der Hoek, 2015; Kuru and Luettgen, 2012; Maimon et al., 2010). Proper treatments are necessary to remove pollutants in greywater, especially microbes. Existing greywater treatments include physical/chemical, biological and ecological processes. Physical/chemical processes are proficient at removing suspended solids but cannot guarantee an adequate reduction of organics and nutrients (Brewer et al., 2001; Gerba et al., 1995; March et al., 2004). The installations of these technologies are relatively inexpensive and easy to operate. Biological processes can ensure satisfactory and stable effluent quality but are relatively complex to operate and expensive to set up (Abdel-Kader, 2013; Liu et al., 2005). Ecological processes, although being the most economical and environment-friendly technologies, commonly require large areas and long storage time that are often not met in urban settings. Comparatively, it is generally agreed that physical/chemical processes are best suited for on-site greywater reuse in most conditions with fair expenses. However, the microbial safety of using greywater treated by physical/chemical processes is one of the most frequently questioned and disputed topics across the literature (De Gisi et al., 2016).

Recent risk analyses associated with greywater on-site irrigation concluded that a well-designed treatment system is required for safe greywater reuse (Maimon et al., 2014). The study also called for a more robust exposure estimation for greywater irrigation in home gardening practices (Maimon et al., 2014). However, the risks from neither consuming homegrown food-crop nor toilet flushing using greywater were investigated previously. Considering both practices are feasible and convenient household applications, we carried out a quantitative microbial risk assessment (QMRA) of greywater on-site reuse for toilet flushing and home garden food-crop irrigation to promote the safe reuse of greywater. The risks were quantified and the implications were discussed.

## 2 Materials and methods

QMRA was carried out following the classical framework that consists of hazard identification, exposure assessment, dose-response assessment and risk characterization (National Research Council, 1983). The Monte Carlo simulation was used to build a probabilistic-based risk model, so that the range and likelihood of the risk were assessed quantitatively. All calculations were conducted using MATLAB R2017a (The MathWorks Inc., Natick, MA).

### 2.1 Hazard identification

#### 2.1.1 Target pathogens in greywater

The potential microbial hazards in domestic greywater have been reported through numerous literature (Burrows et al., 1991; Eriksson et al., 2002; Friedler, 2004). Pathogens including *Salmonella* spp., Norovirus (genogroups GI and GII), Enterovirus, *E. coli*, *Giardia*, *Pseudomonas aeruginosa*, *Staphylococcus aureus*, Clostridia and Rotavirus were detected in greywater from bathrooms, laundries and kitchens in the U. S., England, France, Australia, Hungary and Uganda (Jefferson et al., 2004; Katukiza et al., 2015; Keely et al., 2015; O'Toole et al., 2012). Among all of them, *E. coli* was the most frequently detected and widely distributed potential hazard in most samples (Birks and Hills, 2007; Bodnar et al., 2014; Chaillou et al., 2011; Hargelius et al., 1995; Jefferson et al., 2004; Katukiza et al., 2015; O'Toole et al., 2012; Winward et al., 2008). The existence of pathogenic *E. coli* in domestic greywater, which can cause serious human diseases, was also confirmed by O'Toole et al.'s (2012) study. Therefore, pathogenic *E. coli* was chosen as the target pathogen in the risk analysis.

#### 2.1.2 Pathogenic *E. coli* concentration in greywater

Pathogenic *E. coli* are identified as etiology of various human gastrointestinal illnesses due to the presence of specific colonization factors, virulence factors and pathogenicity associated genes (O'sullivan et al., 2007). Six pathotypes of such strains are now recognized: Verocytotoxigenic *E. coli*, Enterotoxigenic *E. coli*, Enteroinvasive *E. coli*, Enteropathogenic *E. coli*, Enteroaggregative *E. coli* (EAEC) and Diffusely Adherent *E. coli*.

Due to the complexity of methods for quantitative determination of pathogenic *E. coli*, direct measurements of their concentration in greywater were rare in previous studies. Only O'Toole et al. (2012) reported the detection of virulence gene markers among *E. coli* isolates but gave no concentration of pathogenic *E. coli*. In consideration of the data availability, data of total *E. coli* concentration in domestic greywater were collected instead. A pathogenic ratio was introduced to estimate concentration of pathogenic *E. coli* based on total *E. coli* data using,

(1) $$C_{\mathrm{PEC}}=C_{\mathrm{EC}}\times R_{\mathrm{path}}$$

where *C*_*PEC*_ is the estimated concentration of pathogenic *E. coli* in domestic greywater (CFU/100 ml), *C*_*EC*_ is the measured concentration of *E. coli* in domestic greywater (CFU/100 ml), and R_path_ is the pathogenic ratio from *E. coli* to pathogenic *E. coli* (unitless). The pathogenic ratio was calculated as the proportion *E. coli* that are positive for target toxin genes in all *E. coli* isolates tested according to O'Toole et al.'s (2012) result. In view of the uncertainty of the estimation, the worst-case scenario (*R*_*path*_ = 1), in which all *E. coli* detected were assumed to be pathogenic, was also taken into account through the risk assessment.

#### 2.1.3 Probability distribution fit for *E. coli* data

Data of *E. coli* concentration in greywater from different domestic sources are presented in Table 1. As for greywater from bathroom, means and standard deviations of *E. coli* concentration, in units of CFU/100 ml or log_10_CFU/100 ml, were obtained from six previous studies. *E. coli* concentration data from each study was assumed to follow a unimodal log_10_-transformed normal distribution because most microbial and environmental measurement data are distributed log-normally (Hirano et al., 1982; Loper et al., 1984).

**Table 1:** Summary of *E. coli* concentrations in domestic greywater collected from literature.

Source category	Specific sources	Reference	No. of samples	*C*_*EC*_((CFU/100ml)		log_10_*C*_*EC*_ (log_10_CFU/100ml)	
				Mean	Standard deviation	Mean	Standard deviation
Bath-room	Shower/Bath^a^	Bodnar et al. (2014)	30			3.3^c^	2.30^c^
	Shower & Washing	Birks and Hills (2007)	28	3.9E+05^c^	2.4E+06^c^	4.8^d^	1.26^d^
	Shower & Washing	Winward et al. (2008)	54			2.8^c^	0.80^c^
	Shower & Washing	Chaillou et al. (2011)	5	4.8E+05^c^	9.0E+05^c^	5.3^d^	0.81^d^
	Shower & Washing	O'Toole et al. (2012)	36	1.7E+03^c^	4.5E+03^c^	2.8^d^	0.95^d^
	Shower & Washing	Katukiza et al. (2015)	27	6.1E+06^c^	7.6E+05^c^	6.8^d^	0.08^d^

Laundry	Washing machine wash^b^	O'Toole et al. (2012)	75	1.1E+05^c^	9.5E+05^c^	4.1^d^	1.37^d^
	Washing machine rinse^b^	O'Toole et al. (2012)	74	3.4E+03^c^	8.8E+02^c^	3.5^d^	0.17^d^
	Laundry	Bodnar et al. (2014)	30			2.5^c^	2.30^c^
	Laundry	Katukiza et al. (2015)	27	3.7E+06^c^	2.5E+05^c^	6.6^d^	0.04^d^

Kitchen	Kitchen	Hargelius et al. (1995)	4	Observed values: 2.85, 6.60, 6.62, 8.83 log_10_CFU·100 ml^-1^			

a Data were considered the same as ‘Shower & Washing’.

b The way in which these data were used is described in Section 2.1.3.

c Values were generated directly from literature.

d According to the log-normal assumption, the location (μ) and scale (σ) parameters can be obtained if the arithmetic mean and the arithmetic variance are known (Appendix A).

**Fig. 1:**
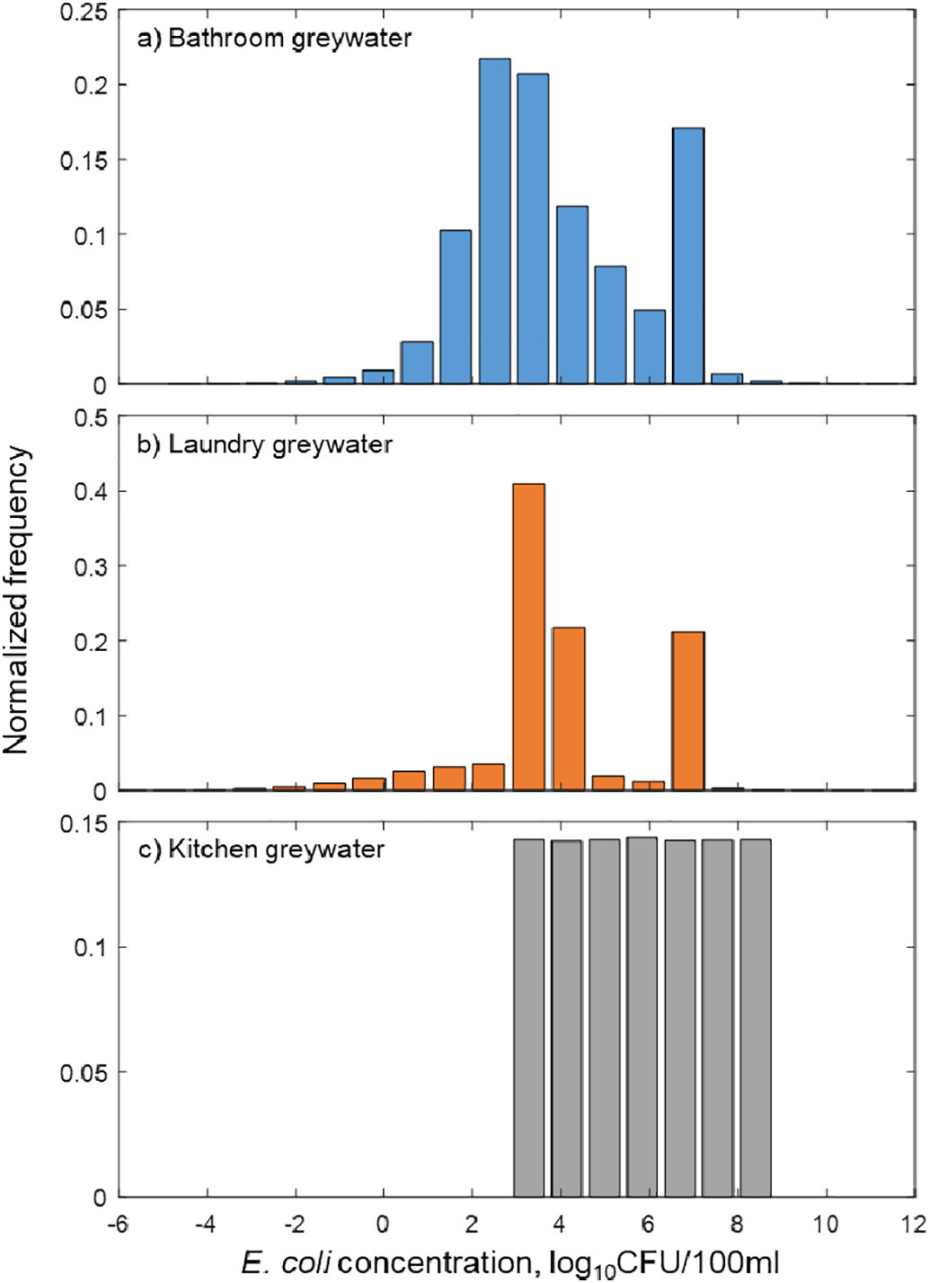
Distribution of log_10_*E. coli* concentration in greywater from residential uses.

The log_10_-transformed concentrations (Table 1) were adopted to build a multimodal normal distribution for bathroom greywater, in which sample number of each study was regarded as weight value. The distribution is shown in Fig. 1a, and the fitting parameters are presented in Table 2.

**Table 2:** List of parameters used in hazard identification.

Description	Symbol	Unit	Point estimate	Probability distribution	Reference
Pathogenic ratio of *E. coli* in bathroom greywater	*R*_*path*, *bat*_	Unitless	0.028 or 1		O'Toole et al. (2012)
Pathogenic ratio of *E. coli* in laundry greywater	*R*_*path*, *lau*_	Unitless	0.027 or 1		
Pathogenic ratio of *E. coli* in kitchen greywater	*R*_*path*, *kit*_	Unitless	0.028^a^ or 1		
Log_10_*E. coli* concentration in greywater from bathroom	lo*g*_10_*C*_*EC*, *bat*_	log_10_CFU/100ml		0.17 × N(3.3, 2.3) + 0.15 × N(4.8, 1.3) + 0.3 × N(2.8, 0.80) +0.03 × N(5.3, 0.81) + 0.20 × N(2.8, 0.95) + 0.15 × N(6.8, 0.08)	
Log_10_*E. coli* concentration in greywater from laundry	lo*g*_10_*C*_*EC*, *lau*_	log_10_CFU/100ml		0.56 × [0.17 × N(4.1, 1.4) + 0.83 × N(3.5, 0.17)] +0.23 × N(2.5, 2.3) + 0.21 × N(6.6, 0.04)	
Log_10_*E. coli* concentration in greywater from kitchen	lo*g*_10_*C*_*EC*, *kit*_	log_10_CFU/100ml		Uniform(2.85, 8.83)	
Greywater volumes from washing machine wash & rinse^b^	*V*_*wash*_*V*_*rinse*_	liters/capita/day	2.26, 10.74		Friedler (2004)
Log_10_ reduction of *E. coli* by microfiltration	*Log*_*MF*_	log_10_CFU/100ml	4		Till et al. (1998), Zheng et al. (2005)

a Data of kitchen greywater was not available, the greater value of bathroom and laundry greywater was adopted as an estimation.

b Volumes were not available in O’Toole’s study, so a study conducted in the same country was referred to.

Similarly, the *E. coli* data of laundry greywater from previous studies were used to build the probability distribution of *E. coli* concentration (Fig. 1b), except those from O'Toole's study with wash water and rinse water separately reported. A bimodal log_10_-transformed normal distribution was used to integrate these data before they were combined with those from other studies. The volume for each step of laundry was used as weight value (Table 2).

Only four observed values were available for greywater from kitchen (Table 1). Due to the deficiency of data and the existence of various food residues in kitchen greywater, which could lead to considerable uncertainty (De Gisi et al., 2016; Friedler, 2004), a log_10_-transformed uniform probability distribution was applied to estimate *E. coli* concentration (Fig. 1c). The minimum and maximum values were adopted as two boundaries (Table 2).

#### 2.1.4 Removal rate by treatment process

To account for the likely on-site treatment before reuse, a physical treatment process – microfiltration was selected as the treatment process for greywater reuse in this study because of its pervasiveness, simplicity and low cost. A 4-log_10_ *E. coli* reduction value was allocated to the microfiltration according to previous experimental results on the removal rate for *E. coli* (Till et al., 1998; Zheng et al., 2005).

### 2.2 Exposure assessment

#### 2.2.1 Toilet-flushing scenario

The main ingestion route of pathogenic *E. coli* through toilet-flushing scenario is inhalation of splashed greywater aerosols. Since pathogenic *E. coli* mainly cause gastrointestinal infection, only aerosols led to gastrointestinal tracts were taken into account. An assumption was made in this study that all aerosols trapped by human noses are cleared to gastrointestinal tracts to represent a worst-case scenario. This assumption is based on previous reports of human breathing pattern and pathogen ingestion mode through aerosols (Couch et al., 1966; Fry and Black, 1973; Stuart, 1984).

Concentration of aerosols in various diameter sizes at different heights in the air after each toilet flush were measured by O'Toole et al. (2009). Data collected at a sampling height of 420 mm above toilet, after a full flush (9 L) were adopted for the analysis. The sampling height here represents a reasonable scenario that an adult is bending down to flush the toilet or it's a small child who is flushing the toilet.

The inhalation efficiency of aerosols was considered on basis of individuals' breathing pattern during light activities (Moya et al., 2011). A breathing rate of 15 l of air/min, obtained from a breathing cycle period equals 8 s (4 s each for inspiration and expiration) and a 1 -l of tidal volume for each cycle, was adopted. The deposition efficiencies of aerosols in extrathoracic (nasal and laryngeal) region were derived from Heyder et al.'s (1986) study, which was reported as a function of particle size and breathing patterns. Furthermore, a distinction was made between nasal and oral breathing, as they could result in totally different deposition efficiencies of aerosols in extrathoracic region (Stuart, 1984). The exposed duration that refers to time spent in toilet room after one flushing, was set at a typical value of 1 min and a worst-case value of 5 min to represent diverse situations. All values and references of parameters mentioned above are listed in Table 3.

**Table 3:** List of parameters used in exposure assessment.

Description		Symbol	Unit	Point estimate		Probability distribution	Reference
**Toilet flushing scenario**							
Concentration of aerosols in air after one toilet flushing		*C*_*aero*, *diam*_*i*__	# of aerosol/l of air				O'Toole et al. (2009)
Median diameter size, i	0.6 μm					Uniform(0, 1.07E+05)	
	2.5 μm					Uniform(0, 1.16E+04)	
Deposition efficiency of aerosols in extrathoracic region		*DE*_*diam*_*i*__	Unitless	Oral/Nasal Breathing			Heyder et al. (1986)
Median diameter size of aerosols, i	0.6 μm			0	0.04		
	2.5 μm			0.01	0.42		
Mean flow rate during human breathing		*MFR*_*air*_	-l of air/min	15			Moya et al. (2011)
Time spent in restroom after one toilet flushing		*T*_*toilet*_	min/flush	Mean: 1 Worst-case: 5			

**Food-crop irrigation scenario**							
Environmental decay rate of *E. coli* on lettuce		log_*decay*_	log_10_/day	0.22			Sjølander (2012)
Withholding time (between last irrigation and eating)		T_*withhold*_	days			Uniform(0, 3)	
Lettuce intake rate per unit body weight per day		*R*_*lettuce*_	g of lettuce/kg/day			Empirical distribution from data reported	Moya and Phillips (2001)
Body weight of U.S. population		*M*_*body*_	kg			Empirical distribution from data reported	Kahn and Stralka (2009)
Volume of water retained on per unit weight of lettuce		*V*_*retention*_	100ml/g of lettuce			Uniform(2.4E-05, 4.8E-05)	

The dose of pathogenic *E. coli* (*Dose*_*PEC*, *toilet*_) inhaled and deposited in human gastrointestinal system (in CFU/flush) after each toilet flushing was estimated as:

(2) $$D{\mathrm{ose}}_{\mathrm{PEC},\mathrm{toilet}}=\sum_{i=1}^{n}C_{\mathrm{PEC},\mathrm{treated}}\times {\mathrm{AerosolDose}}_{{\mathrm{diam}}_{i}}\times {\mathrm{MFR}}_{\mathrm{air}}\times T_{\mathrm{toilet}}=\left(C_{\mathrm{PEC}}\times 10^{-\log_{\mathrm{MF}}}\right)\times \left[\sum_{i=1}^{n}\left(C_{\mathrm{aero},{\mathrm{diam}}_{i}}\times V_{\mathrm{aero},{\mathrm{diam}}_{i}}\times {\mathrm{DE}}_{{\mathrm{diam}}_{i}}\right)\right]\times {\mathrm{MFR}}_{\mathrm{air}}\times T_{\mathrm{toilet}},$$

where *C*_*PEC*, *treated*_ is the concentration of pathogenic *E. coli* in treated greywater (CFU/100 ml), *AerosolDose*_*diam*_*i*__ is the mass of water aerosol (according to median diameter size, i) deposited in extrathoracic region (g/min), *MFR*_*air*_ is the mean flow rate of air breathed after toilet flushing (l of air/min), *T*_*toilet*_ is the time spent in the toilet room after each toilet flushing (min/flush), log_*MF*_ is the log_10_ reduction rate of pathogenic *E. coli* by microfiltration (unitless), *C*_*aero*, *diam*_*i*__ is the concentration of aerosols in the air splashed after toilet flushing (# of aerosol/-l of air), *V*_*aero*, *diam*_*i*__ is the volume of spherical aerosol (100 ml/aerosol) and *DE*_*diam*_*i*__ is the deposition efficiency of aerosols in extrathoracic region (unitless).

#### 2.2.2 Food-crop irrigation scenario

The transfer of pathogens from treated greywater to human body happens when greywater irrigated edible portion of home produce are eaten raw. Such food crops are well-recognized vectors for foodborne diseases (Berger et al., 2010; Olaimat and Holley, 2012), including salad greens, tomatoes, lettuce, cucumber, and pepper. Lettuce was modeled as the representative vegetable in this study due to its popularity as salad green and its propensity to cause human diseases from surface contamination (Lim and Jiang, 2013).

Water retention rate on the surface of lettuce determines the intake rate of contaminants carried in greywater when lettuce is ingested raw. The water adsorption on lettuce using laboratory experiments reported by Shuval et al. (1997) was adopted in the exposure model (Table 3). Water sprays generated during irrigation may represent another pathogen inhalation pathway. However, due to the low exposure volume through water spray inhalation in comparison to food ingestion, the inhalation volume was not included in the assessment. This assumption is further justified by the low probability of human contact during irrigation because most of the spray irrigation in the U.S. occurs at night or early morning hours to reduce transevaporation (National Academies of Sciences and Medicine, 2016).

It's assumed that home irrigated lettuce is watered every three days, and the environmental decay of *E. coli* deposited on surface of lettuce occurs between adjacent irrigations. Therefore, a uniform distribution with boundaries of 0 and 3 days was used to estimate withholding time between last irrigation and consumption of lettuce. The inactivation rate of *E. coli* on lettuce was derived from Sjølander's (2012) study on wastewater irrigated vegetables, which represented a very similar scenario to that used in this study.

The daily intake of lettuce was calculated as a product of human body weight and lettuce intake rate, where the intake rate was expressed as grams of lettuce per kg body weight per day (g of lettuce/kg/day). Empirical distributions of lettuce intake rate and US population' body weight were established from percentile values of survey data reported by previous research (Kahn and Stralka, 2009; Moya and Phillips, 2001) (Table 3).

The dose of pathogenic *E. coli* (Dose_PEC, foodcrop_) ingested through intake of raw lettuce (in CFU/day) was estimated as

(3) $${\mathrm{Dose}}_{\mathrm{PEC},\mathrm{foodcrop}}=C_{\mathrm{PEC},\mathrm{treated}}\times 10^{-\log_{\mathrm{decay}}\times T_{\mathrm{withhold}}}\times R_{\mathrm{lettuce}}\times M_{\mathrm{body}}\times V_{\mathrm{retention}}$$

where log_*decay*_ is the log_10_ environmental decay rate of *E. coli* on lettuce (log_10_/day), *T*_*withhold*_ is the duration of environmental decay (days), *R*_*lettuce*_ is the mass of raw lettuce intake per unit body weight per day (g of lettuce/kg/day), *M*_*body*_ is the body weight of U.S. population (kg), and *V*_*retention*_ is the volume of water retained on per unit weight of lettuce (100 ml/g of lettuce).

### 2.3 Dose-response assessment

The infection or illness risk is commonly expressed as per person per day based on the dose of daily exposure to pathogens. It should be noted that, however, infection includes cases with either symptomatic (showing clinical signs of illness) or asymptomatic (not showing clinical signs of illness) features, while illness only refers to symptomatic cases.

Given a known dose of pathogen, dose-response models, which are generated based on clinical trial data, were used to estimate the risk of a response (e.g. infection or illness). As for pathogenic *E. coli*, the most widely accepted dose-infection model is characterized as a beta-Poisson model (DuPont et al., 1971),

(4) $$P_{\inf}=1-{\left[1+\mathrm{Dose}\frac{2^{\frac{1}{\alpha }}-1}{N_{50}}\right]}^{-\alpha }$$

where P_inf_ is the estimated infection risk, Dose represents the dose of pathogenic *E. coli* ingested (CFU), α and *N*_50_ are best-fit parameters of the model (Table 4).

**Table 4:** List of parameters used in dose-response assessment and risk characterization.

Description	Symbol	Unit	Point estimate	Reference
**Dose-response assessment**				
Parameters for dose-infection model	*α*	-	0.155	DuPont et al. (1971)
	*N*_50_		2.11E+06	
Parameter for dose-illness model	*k*		1.22E-08	

**Risk characterization**				
Times of toilet flushing in one day	Freq_flush_	Times	8	
Times of eating lettuce in one day	Freq_foodcrop_	Times	1	
DALYs per illness case caused by pathogenic *E. coli*	*DALYs*/illness case_*PEC*_	DALYs per illness case	0.0455	Havelaar et al. (2015)

The dose-illness model is characterized by an exponential function (DuPont et al., 1971),

(5) $$P_{\mathrm{ill}}=1-\exp\left(-k\times \mathrm{Dose}\right)$$

where P_ill_ is the estimated illness risk, and *k* is the best-fit parameter of the model which represents the pathogenicity of pathogenic *E. coli*.

The parameter values used in dose-response model are listed in Table 4.

### 2.4 Risk characterization

The acceptable annual infection risk level proposed by the EPA (2005) and the acceptable disability-adjusted life years (DALYs) proposed by WHO (2008) are two most authoritative and widely-used health risk benchmarks. The U.S. EPA benchmark is ≤10^−4^ infection cases pppy, while the WHO benchmark is ≤10^−6^ DALYs pppy.

The annual infection and illness risk were calculated based on the theorem of independence using (Haas et al., 1999)

(6) $$P_{\inf,{\text{annual}}_{\text{scenario}}}=\prod_{i=1}^{n=365\times {\mathrm{Freq}}_{\mathrm{scenario}}}\left(1-P_{\inf}\right),$$

(7) $$P_{\mathrm{ill},{\text{annual}}_{\text{scenario}}}=\prod_{i=1}^{n=365\times {\mathrm{Freq}}_{\mathrm{scenario}}}\left(1-P_{\mathrm{ill}}\right),$$

where P_inf, annual_scenario__ and P_ill, annual_scenario__ are the estimated annual infection risk and annual illness risk, Freq_scenario_ is the number of times a certain scenario occurs during a day, and *n* represents the total number of occurrence of the scenario in a year.

For toilet flushing scenario, a frequency of 8 times a day was applied to represent the worst-case scenario of a healthy human. The frequency of consumption of greywater irrigated lettuce was set to once a day, because the *Dose*_*PEC*, *foodcrop*_ described in Section 2.2.2 represents the dose of pathogenic *E. coli* ingested through intake of raw lettuce per day.

For annual illness risk, the DALYs was calculated as (Mara et al., 2007)

(8) $${\mathrm{Dose}}_{\mathrm{scenario}}=\frac{\mathrm{DALYs}}{{\text{illness case}}_{\mathrm{PEC}}}\times P_{\inf,{\mathrm{annual}}_{\mathrm{scenario}}}$$

where $\frac{\mathrm{DALYs}}{{\text{illness case}}_{\mathrm{PEC}}}$ is the disability-adjusted life years per illness case caused by pathogenic *E. coli* (Havelaar et al., 2015)(Table 4).

### 2.5 Monte-Carlo simulation

**Fig. 2:**
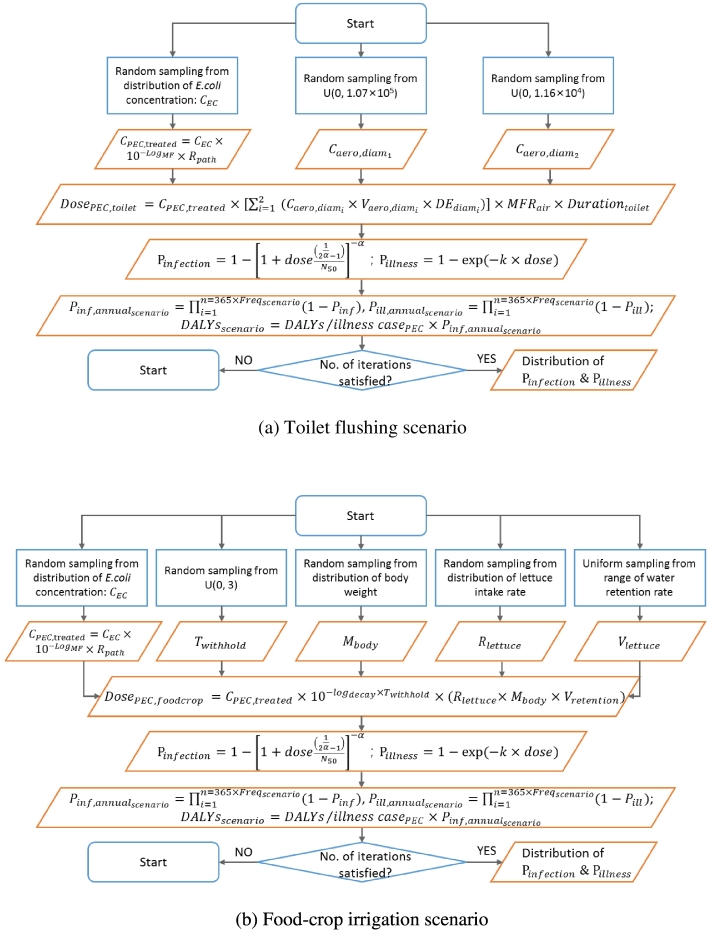
Pseudo-algorithm flow chart for estimating health risks associated with two greywater reuse scenarios.

Monte-Carlo algorithms were written to estimate the probability distribution of microbial risks in both scenarios. Each input parameters (e.g. *E. coli* concentrations, concentration of aerosols in air after each toilet flushing and lettuce intake rate) were randomly selected from their probability distributions. The pseudo-algorithm information flow is shown in Fig. 2. Output parameters (e.g. doses of pathogenic *E. coli* ingested, annual infection risks and disease burdens) were computed over 10,000 iterations so that the distributions can reach a steady state. Small variation (i.e. <1%) in terms of average between replicates of distribution was used for checking reproducibility of model outputs (Lim and Jiang, 2013).

### 2.6 Sensitivity analysis

A local sensitivity analysis was used to assess the variability propagation of each input parameters throughout the risk models (Gottschalk et al., 2010a; Gottschalk et al., 2010b; Manheim and Jiang, 2017). The true means of distributions (or the values of point-estimates) were adopted as baseline point values for each input parameter and output variable. Then a differential value for each output variable X_mean_ was calculated by decreasing the baseline input parameter P_mean_ value by 10% (Gottschalk et al., 2010b). The sensitivities of annual infection risk and disease burden related to each input parameter (e.g. *E. coli* concentration, concentration of aerosols, water retention rate, etc.) were calculated as

(9) $$S=\frac{\left|\Delta X_{\mathrm{mean}}/X_{\mathrm{mean}}\right|}{\left|\Delta P_{\mathrm{mean}}/P_{\mathrm{mean}}\right|}$$

where S is the sensitivity value (unitless), X_*mean*_ is the mean of the output variable distribution using the original values, ΔX_*mean*_ is the difference in means between the original output distribution and the changed output distribution, P_*mean*_ is the mean of the original input distribution (or the value of the original point-estimate), and ΔP_*mean*_ is the difference in means between the original input distribution and the changed input distribution (or the difference between values of original and changed point-estimates). In addition, to identify the most influential contributors to the predicted health risks, all sensitivity values for input parameters were summed to calculate their relative contribution to the total sensitivity.

## 3 Results

### 3.1 Toilet-flushing scenario

**Fig. 3:**
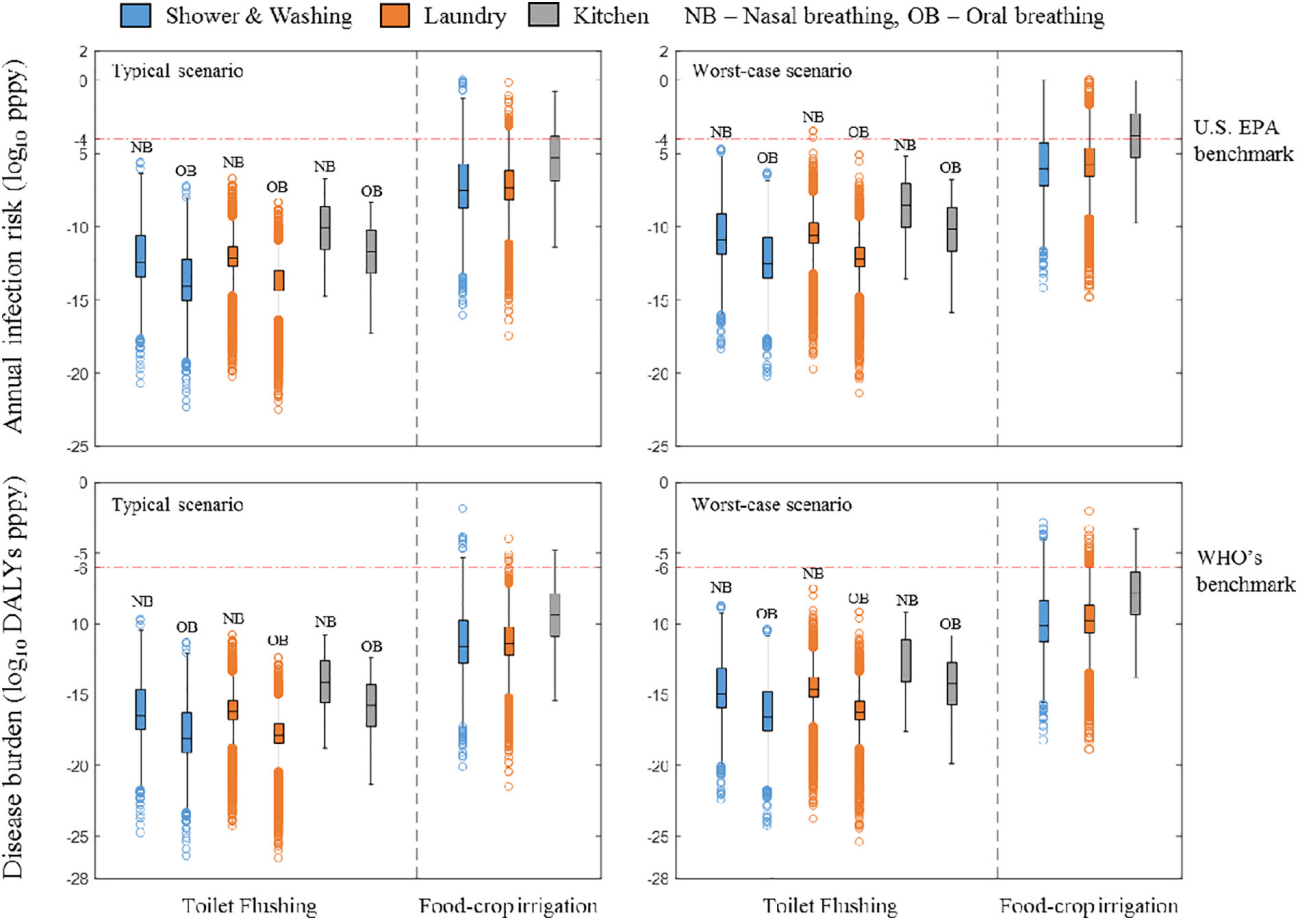
Box-and-Whiskers-Diagram showing annual infection risks and disease burdens from microfiltration-treated greywater on-site reuse^a^. ^a^The bottom and top of the box represents the first and third quartiles (25th & 75th percentile values), while the band inside the box represents the second quartile (median). The whiskers extend 1.5 interquartile range (75th percentile value–25th percentile value) from each end of the box, and markers plotted outside each whisker are considered as outliers.

As shown in Fig. 3, infection risks of pathogenic *E. coli* through toilet flushing using treated domestic greywater are almost negligible. For all scenarios discussed, the infection risks (median range: 8.8 × 10^−15^–8.3 × 10^−11^, 95th percentile range: 2.2 × 10^−11^–4.8 × 10^−8^) are significantly less than the U.S. EPA annual infection benchmark (≤10^−4^ pppy) by orders of magnitude. Even in the worst-case scenario, where all *E. coli* are assumed to be pathogenic, the infection risks are still far below the benchmark.

The infection risks from bathroom greywater and laundry greywater are very close to one another, while the infection risk from kitchen greywater exhibits much higher risk than the former two by approximately two orders of magnitude. The breathing style, as revealed in Fig. 3, plays a similar role, where infection risks for nasal breathers are about 2 log_10_ higher than those for oral breathers. Situations with different durations spent in toilet room after a flush are not specially discussed, because the difference for risk between one-minute and five-minute exposure is within one order of magnitude (Appendix B, Table B.1).

In toilet flushing scenario, a similar conclusion can be made for disease burdens as with infection risks. The values of disease burdens (median range: 7.6 × 10^−19^–7.3 × 10^−15^ DALYs pppy, 95th percentile range: 1.9 × 10^−15^–4.2 × 10^−12^ DALYs pppy) are all far below the benchmark of ≤10^−6^ DALYs pppy proposed by WHO (Fig. 3).

### 3.2 Food-crop irrigation scenario

Infection risks of pathogenic *E. coli* from consuming food-crops irrigated by treated greywater (median range: 2.6 × 10^−8^–4.9 × 10^−6^, 95th percentile range: 1.0 × 10^−4^–4.5 × 10^−3^) are much closer to (or even exceed) the U.S. EPA benchmark (Fig. 3) in comparison with toilet flushing scenarios. Similar to the former application, the infection risk from kitchen greywater is about 2 log_10_ higher than those from bathroom or laundry; the median risk of kitchen greywater (value: 1.7 × 10^−4^) slightly goes beyond 10^−4^ pppy in the worst case and poses a considerable threat for human consumers (Appendix B, Table B.2).

The results of disease burdens present a lower risk, as the values in various cases (median range: 2.3 × 10^−12^–4.3 × 10^−10^, 95th percentile range: 9.1 × 10^−9^–4.0 × 10^−7^) are all below the threshold recommended by WHO. In worst-case scenario, reuse of greywater from bathroom and laundry still exhibit acceptable risks (median: 7.8 × 10^−11^ & 1.4 × 10^−10^, 95th percentile: 4.8 × 10^−7^ & 3.2 × 10^−7^), while the risk from kitchen greywater (median: 1.4 × 10^−8^, 95th percentile: 1.4 × 10^−5^) almost approaches the benchmark.

### 3.3 Un-treated greywater reuse

**Fig. 4:**
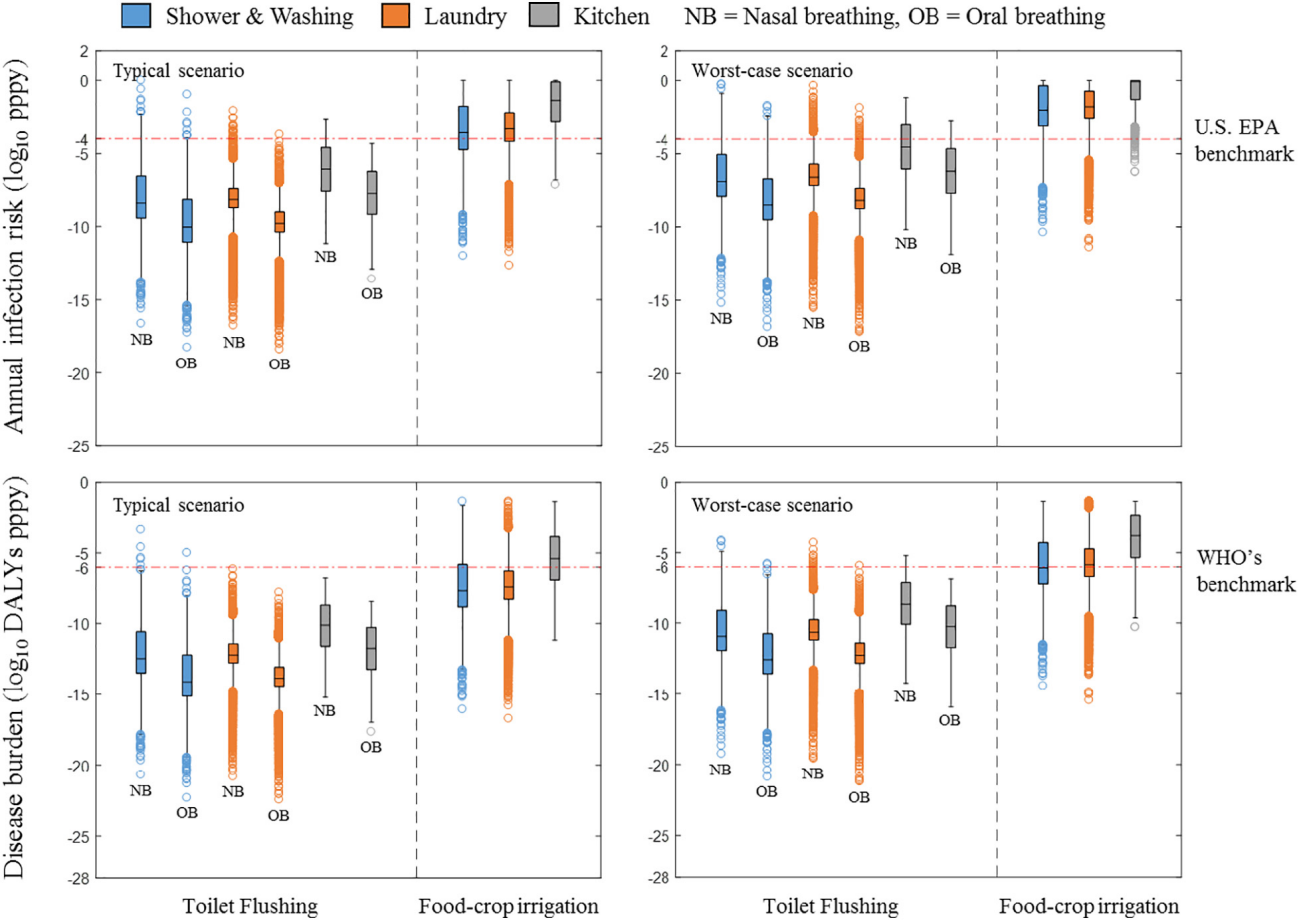
Box-and-Whiskers-Diagram showing annual infection risks and disease burdens from un-treated greywater on-site reuse.

The health risks of reusing un-treated greywater (i.e. the removal rate of pathogenic *E. coli* equals zero) were also estimated as reference values. As shown in Fig. 4, infection risks of pathogenic *E. coli* through toilet flushing using raw domestic greywater are still acceptable. The annual infection risks in all typical scenarios (median range: 8.9 × 10^−11^–8.7 × 10^−7^, 95th percentile range: 3.6 × 10^−7^–4.6 × 10^−5^) satisfy the U.S. EPA annual infection benchmark (≤10^−4^ pppy). But in the worst-case scenario, the infection risks of kitchen greywater (median: 2.8 × 10^−5^ & 6.6 × 10^−7^, 95th percentile range: 1.6 × 10^−2^ & 3.9 × 10^−4^) sometimes exceed the benchmark. As for disease burdens, all values, even the worst-case ones (median range: 2.7 × 10^−13^–2.5 × 10^−9^ DALYs pppy, 95th percentile range: 7.2 × 10^−10^–1.4 × 10^−6^ DALYs pppy), meet the WHO benchmark (Fig. 4).

Annual infection risks of pathogenic *E. coli* from using raw greywater for food-crop irrigation (median range: 2.6 × 10^−4^–4.5 × 10^−2^, 95th percentile range: 0.64–1.0) pose a considerable threat that is far beyond the threshold recommended by the U.S. EPA. Disease burdens in this scenario, although present a relatively lower risk (median range: 2.3 × 10^−8^–4.0 × 10^−6^, 95th percentile range: 9.0 × 10^−5^–3.8 × 10^−3^), are still far from negligible.

### 3.4 Sensitivity analysis

**Fig. 5:**
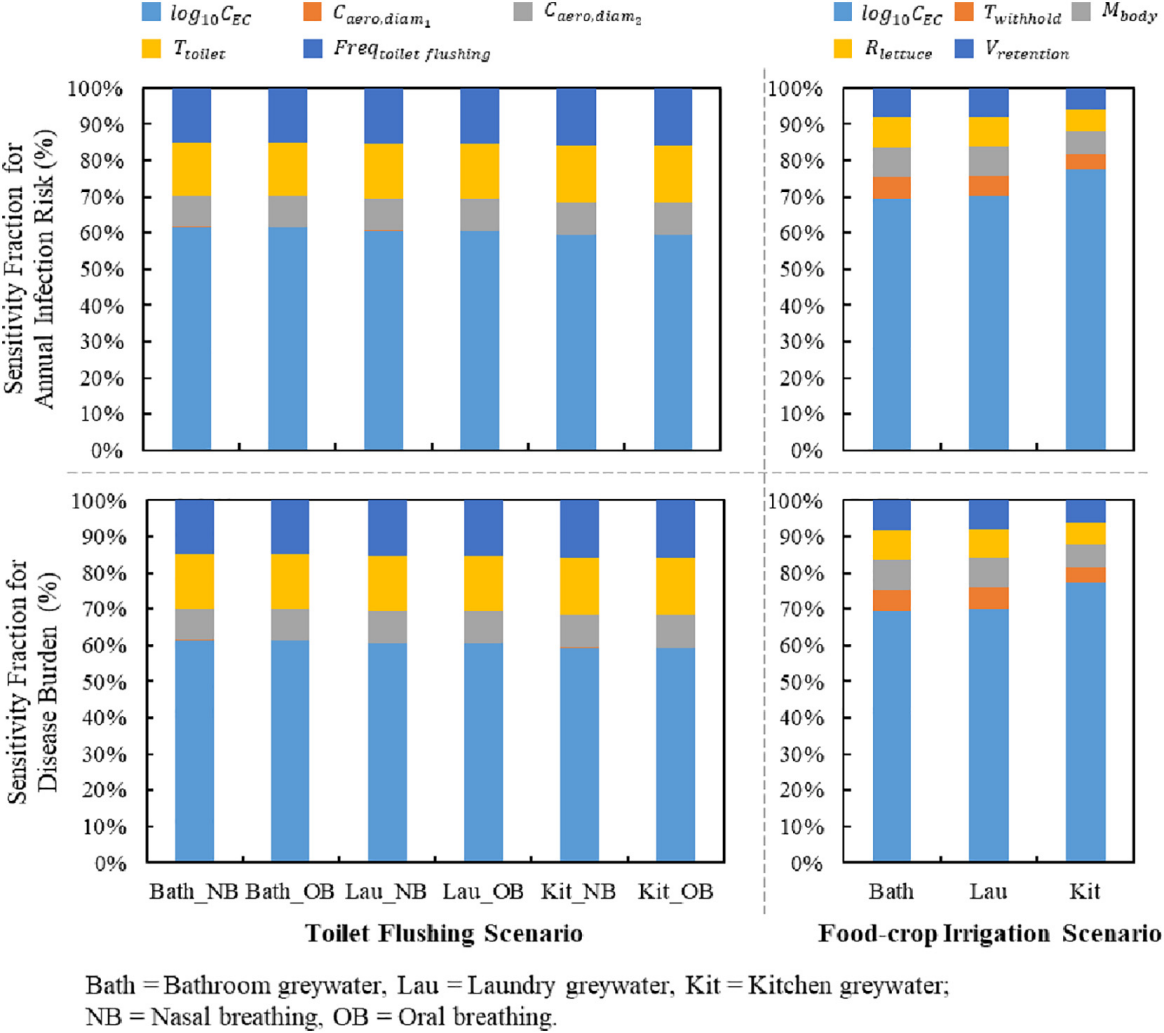
Sensitivity fractions of model input parameters as a function of typical reuse scenarios and greywater sources.

The relative contribution of each input parameters to the variability of infection risks and disease burdens are summarized in Fig. 5. Among the model inputs included in toilet flushing scenario, the output annual infection risks are most sensitive to the input *E. coli* concentration (fraction range: 59.22%–61.51%). Time spent in toilet room (T_*toilet*_) and the daily frequency (Freq_*toiletflushing*_) have the same contributions in each scenario and are far less than that of *E. coli* concentration. Concentrations of aerosols (*C*_*areo*, *diam*_1__, *C*_*areo*, *diam*_2__) are most irrelevant inputs with a combined fraction <10%.

In food-crop irrigation scenario, the input *E. coli* concentration also contributes the highest sensitivity fractions for the output annual infection risks. Especially in the scenario of kitchen greywater reuse, the fraction is nearly 80%. Other input parameters all represent minor contributors to the variability of the infection, with fractions <10%.

## 4 Discussion

### 4.1 Implications

The results of the study showed that greywater food-crop irrigation exhibits a higher health risk than toilet flushing under same conditions. Different greywater sources also lead to disparities between estimated risks. Treated greywater from all three household sources can be used for toilet flushing without significant health risks, while for food-crop irrigation scenario, only greywater from bathroom and laundry can be reused within acceptable risks.

The infection site of the target pathogen may partly explain the low risks associated with toilet flushing scenario. Pathogenic *E. coli* mainly causes gastrointestinal infection, which means the pathogens have to go through the respiratory tract before they ultimately enter their infection site, the digestive tract. Under such condition, only greywater aerosols intercepted in extrathoracic region are possible to be ingested, which result in low exposure doses, and thus low relevant risks. The reason that nasal breathers exhibit a higher risk in this scenario is because of the higher interception efficiency on aerosols. This might be a consequence of the worst-case assumption that all aerosols trapped in extrathoracic region are led to gastrointestinal tracts, since nasal breathing is often considered to be healthier. Future research that provides better understandings of the pathogen ingestion through aerosol inhalation will improve the confidence in this estimate.

Annual infection risk and disease burden are adopted as indicators throughout the analysis. When calculating disease burdens, the unique characteristics of different pathogens on morbidity and mortality are taken into account, which means a pathogen with higher virulence will get a greater DALYs per illness case. In contrast, analyses with annual infection risk regard all pathogens the same important (Gibney et al., 2013). Difference between two indicators could cause sharp disagreement on discussion of risk acceptability.

In this study, only one target pathogen (pathogenic *E. coli*) is used to estimate the health risks, which means all DALYs are calculated by the same dose-illness model. This approach results in the same trends between annual infection risks and disease burdens under the same condition. However, DALYs indicate lower threats than annual infection risks in each scenario according to corresponding benchmarks. This is likely due to the relatively low virulence of pathogenic *E. coli*. It should be noted that, both the WHO's benchmark of ≤10^−6^ DALYs pppy and the U.S. EPA benchmark of ≤10^−4^ infection pppy are established for assessing safety of drinking water. And both are considered overly conservative and impractical for the health risk assessment on non-potable water uses (Mara, 2011; Mara and Sleigh, 2010).

### 4.2 Model uncertainties

#### 4.2.1 Concentration of the target pathogen

Pathogenic *E. coli* is one of the many pathogens in the greywater that may pose considerable risks. Lack of quantitative pathogen data in greywater has been the major hurdle for a comprehensive risk analysis. Probability distribution of *E. coli* concentration in greywater is established based on measured data from various regions of the world (Table 1). The data mining results indicate that concentrations of *E. coli* in bathroom and laundry greywater vary little despite the geographic differences, which certain their reliability in risk estimation in the U.S. In contrast, qualities of kitchen greywater in different societies can vary significantly due to different living habits. For example, Americans are used to directly discarding food residue into kitchen sinks after meals, while Chinese people only use the kitchen sink for washing vegetables and rinse dishes. These household practices can cause considerable difference between kitchen greywater qualities. Since *E. coli* concentration has the greatest influence on risk outcomes based on the sensitivity analysis, additional data on pathogen concentrations in kitchen greywater, especially the local ones, are needed for a more accurate and more reliable health risk assessment.

The pathogenic ratio of *E. coli* used in the model is only calculated by a positive-or-negative detection instead of exact concentrations of pathogenic and total *E. coli* (O'Toole et al., 2012). The credibility of ratio value may contribute to a remarkable uncertainty, the range of which is considered in this study by discussing the worst-case scenario with all *E. coli* being pathogenic and is reducible when improved knowledge available.

#### 4.2.2 Toilet flushing scenario

During toilet flushing, the aerosols generated are proportional to the amount of energy and water volume used in a single flush (Johnson et al., 2013). O'Toole et al. failed to detect any aerosols after flushes with flush volumes of below 4.5 L (O'Toole et al., 2009). Present water-saving toilets are commonly equipped with a full flush volume of 6 L and a half flush of 3 L, which will produce lower aerosol volume and health risks in comparison with the 9 L full flush toilet modeled in this study.

Johnson et al. also reported that the concentration of aerosols drop with the increase of sampling height (Johnson et al., 2013). The aerosol concentration at a location 420 mm above the toilet seat described in Section 2.2.1 is much lower than the common height of a standing adult's nose (or mouth). Considering the short duration of a person to bend down to flush the toilet, aerosols exposed to human noses are likely at lower concentrations than the conservative assumption used in this study.

Furthermore, gravitational shrinkage or sedimentation of aerosols mostly happens within the first 30s after a single flush (Johnson et al., 2013). The dynamic reduction of aerosol concentrations in the air is not included in the model since data are not available. The initial aerosol concentrations are used for the entire exposure as a worst-case estimation. Other factors such human breathing patterns using mix nasal and oral breathing can also influence the risk outcome as indicated in a previous study (Lim et al., 2015).

After all, the aerosol concentrations and the duration of breathing polluted air are not sensitive input parameters for the model. Based on the sensitivity analysis, the model predictions can be afforded relatively high levels of confidence, despite the uncertainties discussed above.

#### 4.2.3 Food-crop irrigation scenario

The health risk result of food-crop irrigation scenario is only fully applicable to the U.S. population because data of body weight and lettuce intake rate used in the model are generated from American domestic surveys. However, since the model outcomes are insensitive to neither of these two parameters, the results are still referable in other regions. Other uncertainties may include pathogen remove from washing lettuce before consumption. In the absence of credible data, a worst-case scenario of no washing before eating is discussed to represent the highest potential risk. Despite that, a thorough washing before eating is highly recommended to dilute possible pathogens retained on produce surface, thus effectively reduce the related health risks (according to the high sensitivity of *E coli.* concentration).

#### 4.2.4 Dose-response models

The most important source of uncertainties, perhaps, is the dose-response models used for estimating the infection or illness probability through a single exposure. Of six known pathotypes of *E. coli*, only EAEC and Enteropathogenic *E. coli* were detected in bathroom greywater and in laundry greywater, respectively (O'Toole et al., 2012). However, due to the lack of relevant studies, pathotypes of *E. coli* in greywater from different sources can't be simply defined. Although the dose-response models of different pathotypes are not exactly the same due to different infection or illness mechanisms, dose-response models have not been established for all (e.g. EAEC) (DuPont et al., 1971; Feeguson and June, 1952; Graham et al., 1983; Haas et al., 1999; June et al., 1953; Levine et al., 1977). All pathotypes of *E. coli* are considered the same, as ‘pathogenic *E. coli*’, in the study; and two most widely accepted dose-response models (dose-infection model and dose-illness model) on pathogenic *E. coli* are used for estimation (Enger et al., 2013; Hayashi, 2016; Weir et al., 2017; Wilkes et al., 2013). A more accurate risk estimation calls for more field study of pathogenic *E. coli* in greywater and clinical infection data of various pathogenic *E. coli* pathotypes.

### 4.3 Contribution and limitation

The interpretation of QMRA results is usually made through comparison with existing water quality standards. For instance, California has adopted a strict microbial standard for toilet flushing using reclaimed water, which requires a 7-day median concentration of ≤2.2 total coliforms/100 ml of water (Lim et al., 2015). Similarly, the standard in China specifies the number of total coliform below 3 per liter of water, which is magnitudes lower than those of German (100 total coliforms/100 ml of reclaimed water) and Japan (1000 total coliforms/100 ml of reclaimed water) (De Gisi et al., 2016). These rules are formulated on basis of different purposes and considerations, but they all intend to make simple yes-or-no judgments by only one (or two) commonly adopted microbial indicator(s) (De Gisi et al., 2016). These over simplified policy decisions are primary due to the consideration of ease for implementation and supervision.

In comparison with applying numerical *E. coli* standards to treated greywater quality, QMRA incorporates type and source of pathogens in specific greywater stream and exposure scenarios to provide the probability distributions of the infection/illness risks. The results of QMRA, which is often regarded as a more pertinent approach for risk characterization, can serve as scientific basis for revision of those standards.

In this study, microfiltration is selected as a typical process to simplify the model. The low health risk of greywater treated by microfiltration can also be generalized for other commonly used membrane processes with smaller pore sizes, such as ultrafiltration, nanofiltration and reverse osmosis. Furthermore, health risks of other treatment processes, such as disinfection used in Aquasave Project (ENEA, 2002), can be estimated using the similar approach, by simply inputting their removal rates (either point estimate or probability distribution) instead of that of microfiltration.

It should be noted that, however, greywater treatment requirement for on-site reuse is beyond health risk concern. For instance, the fouling and odor caused by organics can seriously hinder the practicality of using greywater for toilet flushing (Kuru and Luettgen, 2012). Another threat comes from inorganic salts or surfactants in greywater that might pose great environmental risk to ecosystems through the reuse for irrigating gardens (Lubbe et al., 2016; Pandey et al., 2014). Other basic organoleptic indicators, including color and turbidity, should also be taken into consideration for improving public acceptance of greywater reuse.

**Fig. 6:**
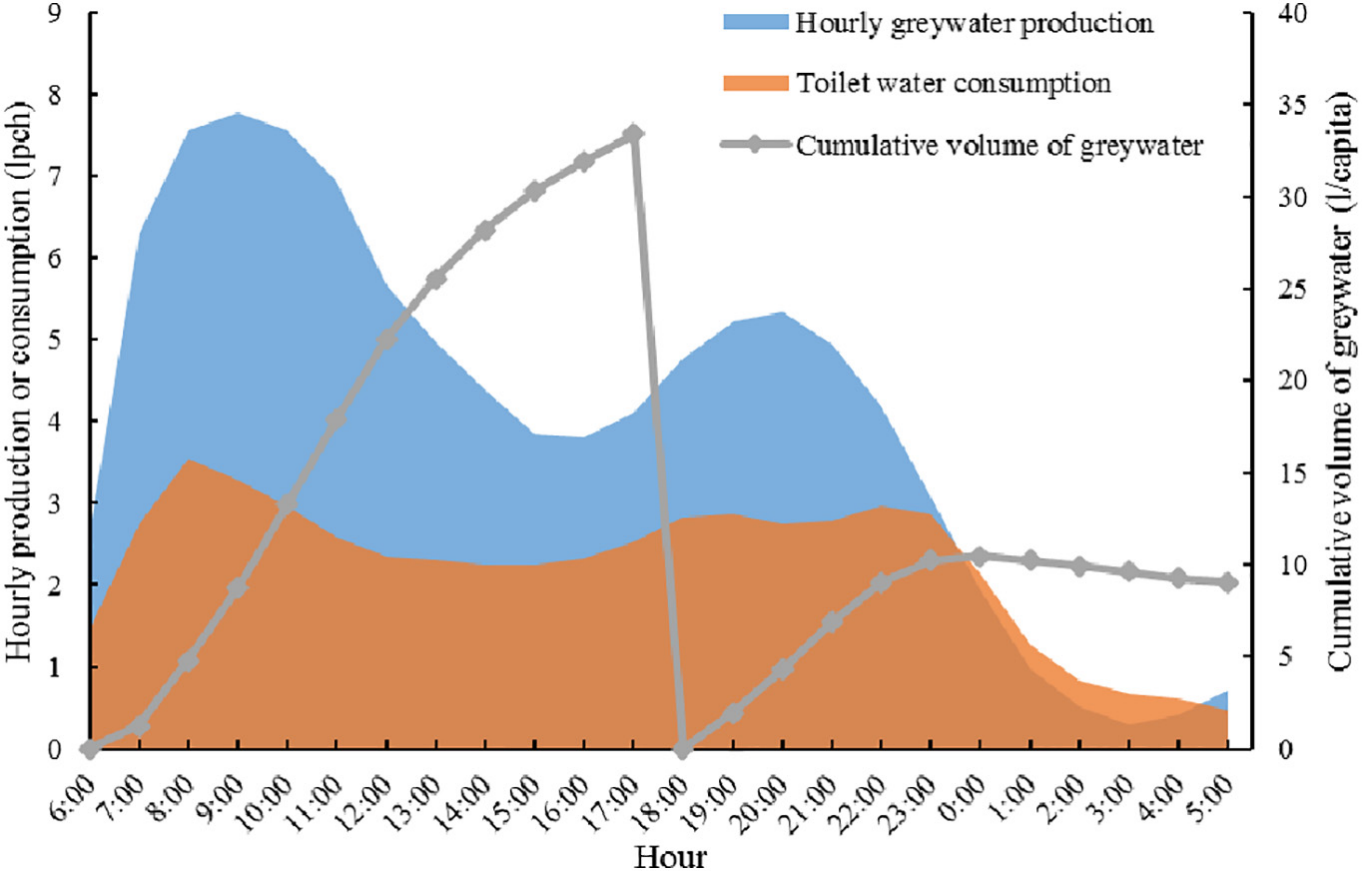
Estimated hourly patterns of greywater production and cumulative volume.

Furthermore, organics and other nutrients in greywater, if not well eliminated, may give rise to regrowth of microorganism when the water is stored in tanks (March et al., 2004). According to the latest data on volumes (DeOreo et al., 2016) and patterns (Mayer et al., 1999) of indoor water uses in a single U.S. household, the hourly greywater production (excludes water from kitchen sink faucets) commonly exceeds the water consumption of toilet flushing (Fig. 6). Assuming all stored greywater in tank are used for outdoor irrigation or drained into the sewer at 6:00 and 18:00 every day (Mayer et al., 1999), there are still greywater accumulation in storage tanks between evacuation.

However, the dynamic process that greywater getting in and out the storage tank makes the estimation of pathogen regrowth far more complicated than a simple time-based growth function. It should also be noted that, only regrowth of indicator microbes (e.g. *E. coli*) was reported in greywater tanks (March et al., 2004), which doesn't necessarily refer to regrowth of real pathogens (e.g. pathogenic *E. coli*). In fact, treated domestic greywater is not often regarded as good environment for pathogen growth. Additional studies are needed to elucidate pathogens' behavior during greywater storage.

## 5 Conclusions

The risk assessment outcomes of using treated household greywater indicate:

• Given the same greywater source, food-crop irrigation exhibits a higher health risk than that of toilet flushing under same conditions.
• Given the same exposure scenario, kitchen greywater poses the highest risk, followed by laundry greywater and bathroom greywater.
• Greywater from bathroom and laundry are safe for both toilet flushing and food-crop irrigation after treated by microfiltration.
• Treated greywater from kitchen is not clean enough for food-crop irrigation while it's innocuous for toilet flushing.
• Many factors contribute to the uncertainties of the risk outcomes. Among them dose-response model and pathogen concentration are the most critical at accuracy of the estimates.
• Overall, greywater on-site reuse should be promoted with proper awareness of the risk.

## Appendix A Supplementary data

Supplementary material Image 1
