# Supplementary material for: Quantitative microbial risk assessment of Greywater on-site reuse
Source: Sci Total Environ. 2018 Sep 1;635:1507–19. doi: 10.1016/j.scitotenv.2018.04.197 (PMC6024565; doi:10.1016/j.scitotenv.2018.04.197)
Supplement: Supplementary file 1 — Supplementary material [file mmc1.doc]

## Appendix A. Deduction of log-normal distribution parameters

Given that: X has a normal distribution; μ and σ are mean and standard deviation of log_10_X, respectively; E(X) and Var(X) are the arithmetic mean and arithmetic standard deviation of X, μ and σ can be obtained if the arithmetic mean and the arithmetic variance can be calculated as:

$\mu=\text{l}og_{10}(\frac{E(X)^{2}}{\sqrt{E(X)^{2}}})=\log_{10}(\frac{E(X)^{2}}{\sqrt{Var(X)+E(X)^{2}}})$ (A-1)

$\sigma^{2}=\text{l}og_{10}(\frac{E(X^{2})}{E(X)^{2}})=\log_{10}(1+\frac{Var(X)}{E(X)^{2}})$ (A-2)

## Appendix B. Summary tables for risk assessment results

Table B.1: Summary descriptors of risk assessment for toilet flushing scenario (treated greywater)

| **Toilet flushing** |  | Median | | | | | | 95^th^ percentile | | | | | |
| --- | --- | --- | --- | --- | --- | --- | --- | --- | --- | --- | --- | --- | --- |
|  |  | Shower & Washing | | Laundry | | Kitchen | | Shower & Washing | | Laundry | | Kitchen | |
|  | 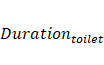 | Nasal | Oral | Nasal | Oral | Nasal | Oral | Nasal | Oral | Nasal | Oral | Nasal | Oral |
| Annual infection risk (Typical: $R_{path}<1$ ) | 1 min | 3.8E-13 | 8.8E-15 | 6.7E-13 | 1.6E-14 | 8.3E-11 | 1.9E-12 | 1.5E-09 | 3.4E-11 | 9.3E-10 | 2.2E-11 | 4.8E-08 | 1.1E-09 |
|  | 5 min | 1.9E-12 | 4.4E-14 | 3.4E-12 | 7.9E-14 | 4.2E-10 | 9.5E-12 | 7.2E-09 | 1.7E-10 | 4.7E-09 | 1.1E-10 | 2.4E-07 | 5.7E-09 |
| Annual infection risk (Worst: ) | 1 min | 1.4E-11 | 3.2E-13 | 2.5E-11 | 5.9E-13 | 3.1E-09 | 7.0E-11 | 5.4E-08 | 1.3E-09 | 3.5E-08 | 8.2E-10 | 1.7E-06 | 4.0E-08 |
|  | 5 min | 6.9E-11 | 1.6E-12 | 1.3E-10 | 3.0E-12 | 1.5E-08 | 3.5E-10 | 2.7E-07 | 6.3E-09 | 1.7E-07 | 4.1E-09 | 8.4E-06 | 2.0E-07 |
| DALYs  (Typical: ) | 1 min | 3.3E-17 | 7.6E-19 | 5.8E-17 | 1.4E-18 | 7.3E-15 | 1.7E-16 | 1.3E-13 | 3.0E-15 | 8.1E-14 | 1.9E-15 | 4.2E-12 | 9.9E-14 |
|  | 5 min | 1.6E-16 | 3.8E-18 | 2.9E-16 | 6.9E-18 | 3.6E-14 | 8.3E-16 | 6.3E-13 | 1.5E-14 | 4.1E-13 | 9.6E-15 | 2.1E-11 | 5.0E-13 |
| DALYs  (Worst: ) | 1 min | 1.2E-15 | 2.8E-17 | 2.2E-15 | 5.1E-17 | 2.7E-13 | 6.1E-15 | 4.7E-12 | 1.1E-13 | 3.0E-12 | 7.2E-14 | 1.5E-10 | 3.5E-12 |
|  | 5 min | 6.1E-15 | 1.4E-16 | 1.1E-14 | 2.6E-16 | 1.3E-12 | 3.0E-14 | 2.3E-11 | 5.6E-13 | 1.5E-11 | 3.6E-13 | 7.4E-10 | 1.7E-11 |

Table B.2: Summary descriptors of risk assessment for food-crop irrigation scenario (treated greywater)

| **Food-crop irrigation** |  | Median | | | 95^th^ percentile | | |
| --- | --- | --- | --- | --- | --- | --- | --- |
|  | 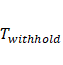 | Shower & Washing | Laundry | Kitchen | Shower & Washing | Laundry | Kitchen |
| Annual infection risk (Typical: ) | U(0, 3) day | 2.6E-08 | 4.9E-08 | 4.9E-06 | 1.6E-04 | 1.0E-04 | 4.5E-03 |
|  | 0 day | 5.4E-08 | 1.0E-07 | 1.1E-05 | 3.4E-04 | 2.2E-04 | 8.7E-03 |
|  | 3 day | 1.2E-08 | 2.2E-08 | 2.4E-06 | 7.4E-05 | 4.8E-05 | 1.9E-03 |
| Annual infection risk (Worst: ) | U(0, 3) day | 8.9E-07 | 1.7E-06 | 1.7E-04 | 5.5E-03 | 3.7E-03 | 1.5E-01 |
|  | 0 day | 1.9E-06 | 3.6E-06 | 3.5E-04 | 1.2E-02 | 8.0E-03 | 2.7E-01 |
|  | 3 day | 4.2E-07 | 7.8E-07 | 7.6E-05 | 2.5E-03 | 1.8E-03 | 6.7E-02 |
| DALYs  (Typical: ) | U(0, 3) day | 2.3E-12 | 4.2E-12 | 4.3E-10 | 1.4E-08 | 9.1E-09 | 4.0E-07 |
|  | 0 day | 4.7E-12 | 8.9E-12 | 9.5E-10 | 2.9E-08 | 1.9E-08 | 7.6E-07 |
|  | 3 day | 1.0E-12 | 2.0E-12 | 2.1E-10 | 6.4E-09 | 4.2E-09 | 1.7E-07 |
| DALYs  (Worst: ) | U(0, 3) day | 7.8E-11 | 1.4E-10 | 1.4E-08 | 4.8E-07 | 3.2E-07 | 1.4E-05 |
|  | 0 day | 1.7E-10 | 3.1E-10 | 3.0E-08 | 1.0E-06 | 7.1E-07 | 2.8E-05 |
|  | 3 day | 3.7E-11 | 6.8E-11 | 6.6E-09 | 2.2E-07 | 1.5E-07 | 6.1E-06 |

Table B.3: Summary descriptors of risk assessment for toilet flushing scenario (non-treated greywater)

| **Toilet flushing** |  | Median | | | | | | 95^th^ percentile | | | | | |
| --- | --- | --- | --- | --- | --- | --- | --- | --- | --- | --- | --- | --- | --- |
|  |  | Shower & Washing | | Laundry | | Kitchen | | Shower & Washing | | Laundry | | Kitchen | |
|  | 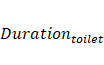 | Nasal | Oral | Nasal | Oral | Nasal | Oral | Nasal | Oral | Nasal | Oral | Nasal | Oral |
| Annual infection risk (Typical: ) | 1 min | 3.8E-09 | 8.9E-11 | 6.8E-09 | 1.6E-10 | 8.7E-07 | 2.0E-08 | 1.5E-05 | 3.6E-07 | 9.3E-06 | 2.2E-07 | 4.6E-05 | 1.1E-05 |
|  | 5 min | 1.9E-08 | 4.5E-10 | 3.4E-08 | 7.9E-10 | 4.4E-06 | 9.9E-08 | 7.6E-05 | 1.8E-06 | 4.6E-05 | 1.1E-06 | 2.4E-03 | 5.5E-05 |
| Annual infection risk (Worst: ) | 1 min | 1.3E-07 | 3.0E-09 | 2.6E-07 | 6.0E-09 | 2.8E-05 | 6.6E-07 | 5.4E-04 | 1.3E-05 | 3.5E-04 | 8.2E-06 | 1.6E-02 | 3.9E-04 |
|  | 5 min | 6.5E-07 | 1.5E-08 | 1.3E-06 | 3.0E-08 | 1.4E-04 | 3.3E-06 | 2.7E-03 | 6.3E-05 | 1.7E-03 | 4.1E-05 | 7.9E-02 | 1.9E-03 |
| DALYs  (Typical: ) | 1 min | 3.3E-13 | 7.8E-15 | 5.9E-13 | 1.4E-14 | 7.6E-11 | 1.7E-12 | 1.3E-09 | 3.1E-11 | 8.1E-10 | 1.9E-11 | 4.1E-08 | 9.7E-10 |
|  | 5 min | 1.7E-12 | 3.9E-14 | 2.9E-12 | 6.9E-14 | 3.8E-10 | 8.7E-12 | 6.6E-09 | 1.6E-10 | 4.0E-09 | 9.6E-11 | 2.1E-07 | 4.8E-09 |
| DALYs  (Worst: ) | 1 min | 1.1E-11 | 2.7E-13 | 2.2E-11 | 5.3E-13 | 2.5E-09 | 5.7E-11 | 4.7E-08 | 1.1E-09 | 3.0E-08 | 7.2E-10 | 1.4E-06 | 3.4E-08 |
|  | 5 min | 5.7E-11 | 1.3E-12 | 1.1E-10 | 2.6E-12 | 1.2E-08 | 2.9E-10 | 2.3E-07 | 5.5E-09 | 1.5E-07 | 3.6E-09 | 7.1E-06 | 1.7E-07 |

Table B.4: Summary descriptors of risk assessment for food-crop irrigation scenario (non-treated greywater)

| **Food-crop irrigation** |  | Median | | | 95^th^ percentile | | |
| --- | --- | --- | --- | --- | --- | --- | --- |
|  | 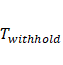 | Shower & Washing | Laundry | Kitchen | Shower & Washing | Laundry | Kitchen |
| Annual infection risk (Typical: ) | U(0, 3) day | 2.6E-04 | 4.7E-04 | 4.5E-02 | 7.9E-01 | 6.4E-01 | 1.0E+00 |
|  | 0 day | 5.6E-04 | 9.9E-04 | 9.3E-02 | 9.6E-01 | 8.9E-01 | 1.0E+00 |
|  | 3 day | 1.2E-04 | 2.2E-04 | 2.1E-02 | 5.2E-01 | 3.9E-01 | 1.0E+00 |
| Annual infection risk (Worst: ) | U(0, 3) day | 9.7E-03 | 1.6E-02 | 8.4E-01 | 1.0E+00 | 1.0E+00 | 1.0E+00 |
|  | 0 day | 2.1E-02 | 3.5E-02 | 9.8E-01 | 1.0E+00 | 1.0E+00 | 1.0E+00 |
|  | 3 day | 4.7E-03 | 7.7E-03 | 2.1E-02 | 1.0E+00 | 1.0E+00 | 1.0E+00 |
| DALYs  (Typical: ) | U(0, 3) day | 2.3E-08 | 4.1E-08 | 4.0E-06 | 1.4E-04 | 9.0E-05 | 3.8E-03 |
|  | 0 day | 4.9E-08 | 8.6E-08 | 8.5E-06 | 2.9E-04 | 2.0E-04 | 7.4E-03 |
|  | 3 day | 1.1E-08 | 1.9E-08 | 1.9E-06 | 6.4E-05 | 4.4E-05 | 1.7E-03 |
| DALYs  (Worst: ) | U(0, 3) day | 8.5E-07 | 1.4E-06 | 4.0E-06 | 4.6E-03 | 3.3E-03 | 3.8E-03 |
|  | 0 day | 1.9E-06 | 3.1E-06 | 8.5E-06 | 9.2E-03 | 6.5E-03 | 7.4E-03 |
|  | 3 day | 4.1E-07 | 6.7E-07 | 1.9E-06 | 2.2E-03 | 1.5E-03 | 1.7E-03 |
